# Supplementary material for: Complex groundwater flow systems as traveling agent models
Source: PeerJ. 2014 Oct 16;2:e557. doi: 10.7717/peerj.557 (PMC4203025; doi:10.7717/peerj.557)
Supplement: Supplemental Information 1 [file peerj-02-557-s001.pdf]

Electronic Supplementary Materials for Complex groundwater flow systems as a traveling agent models

López-Corona O. ,[1] Escolero O. ,[2] Padilla P. ,[3] González T.[4] and Morales E.[2]*

[1]Posgrado en Ciencias de la Tierra, Instituto de Geología, Universidad Nacional Autónoma de México, Circuito Escolar, Cd. Universitaria México D.F.
[2] Instituto de Geología, Universidad Nacional Autónoma de México, Circuito Escolar, Cd. Universitaria México D.F.
[3]IIMAS, Universidad Nacional Autónoma de México, Circuito Escolar, Cd. Universitaria México D.F.
[4] Instituto de Geofísica, Universidad Nacional Autónoma de México, Circuito Escolar, Cd. Universitaria México D.F.
[*]oliverlc@geologia.unam.mx

Derivation of the Flow Equations

Lets consider a group of agents moving in a square lattice according with a strategy e function of actual location and neighbors density. The probability of finding an agent in an arbitrary node $(i\Delta x, j\Delta y)$ at the time $k\Delta t$, as in Figure 1, is

$$P\left(i\Delta x, j\Delta y, k\Delta t\right). \tag{1}$$

The probability of an agent originally in $(i_0\Delta x, j_0\Delta y)$ at the time $k_0\Delta t$ walk to $(i\Delta x, j\Delta y)$ at the next time $k\Delta t = (k+1)\Delta t$ is

$$\begin{array}{rcl} P\left(i\Delta x, j\Delta y, k\Delta t\right) - P\left(i_0\Delta x, j_0\Delta y, k_0\Delta t\right) & = & e^t_{Uij}\left[P\left(i\Delta x, (j+1)\Delta y, k\Delta t\right) - P\left(i_0\Delta x, j_0\Delta y, k_0\Delta t\right)\right] \\ & + & e^t_{Dij}\left[P\left(i\Delta x, (j-1)\Delta y, k\Delta t\right) - P\left(i_0\Delta x, j_0\Delta y, k_0\Delta t\right)\right] \\ & + & e^t_{Rij}\left[P\left((i+1)\Delta x, j\Delta y, k\Delta t\right) - P\left(i_0\Delta x, j_0\Delta y, k_0\Delta t\right)\right] \\ & + & e^t_{Lij}\left[P\left((i-1)\Delta x, j\Delta y, k\Delta t\right) - P\left(i_0\Delta x, j_0\Delta y, k_0\Delta t\right)\right] \end{array} \tag{2}$$

Where e^t_{dij} is the strategy that the agent adopt based on the density neighbors difference between his actual position and the next one in d direction, so

$$\begin{array}{rcl} e^t_{Uij} & = & e\left(\left\|(\nabla P)^t_{i,j+1}\right\|\right) \\ e^t_{Dij} & = & e\left(\left\|(\nabla P)^t_{i,j-1}\right\|\right) \\ e^t_{Rij} & = & e\left(\left\|(\nabla P)^t_{i+1,j}\right\|\right) \\ e^t_{Lij} & = & e\left(\left\|(\nabla P)^t_{i-1,j}\right\|\right) \end{array} \tag{3}$$

If we define $\delta P^t_{Uij} = P\left(i\Delta x, (j+1)\Delta y, k\Delta t\right) - P\left(i_0\Delta x, j_0\Delta y, k_0\Delta t\right)$, and so for the rest of directions, then Eq.2 became

$$P\left(i\Delta x, j\Delta y, k\Delta t\right) = P\left(i_0\Delta x, j_0\Delta y, k_0\Delta t\right) \quad + e^t_{Uij}\delta P^t_{Uij} \quad + e^t_{Dij}\delta P^t_{Dij} + e^t_{Rij}\delta P^t_{Rij} + e^t_{Lij}\delta P^t_{Lij}. \tag{4}$$

Which is the discrete form of the anisotropic diffusion equation[1]

$$\frac{\partial P\left(x, y, t\right)}{\partial t} = div\left[e\left(\|\nabla P\|\right)\nabla P\right]. \tag{5}$$

The discrete anisotropic diffusion equation 4 could be rewritten as[2]

$$P^{t+1}_a = P^t_a + \frac{\lambda}{|\eta_a|}\sum_{b\in\eta_a} e\left(\delta P_{a,b}\right)\delta P_{a,b} \tag{6}$$

with a the actual position, η_s the neighboring position of a, $|\eta_a|$ the number of first neighbors of the the agent in a and $\lambda \in \mathbb{R}^+$ a constant that define the diffusion rate.

Of course, the functional form of e^t_{dij} could be a more generic one as

$$e^t_{dij} = e^t_{dij}\left(x, y, t\right) \tag{7}$$

	C	D
C	R	S
D	T	P

Tab. 1: Canonical payoff matrix for classical Prisoner's dilemma. Where T stands for Temptation to defect, R for Reward for mutual cooperation, P for Punishment for mutual defection and S for Sucker's payoff. To be defined as prisoner's dilemma, the following inequalities must hold: T > R > P > S

in which case the general anisotropic diffusion equation is obtained

$$\frac{\partial P\left(x,y,t\right)}{\partial t}=div\left[e\left(x,y,t\right)\nabla P\right]=\nabla e\cdot\nabla P+e\left(x,y,t\right)\nabla^{2}P. \tag{8}$$

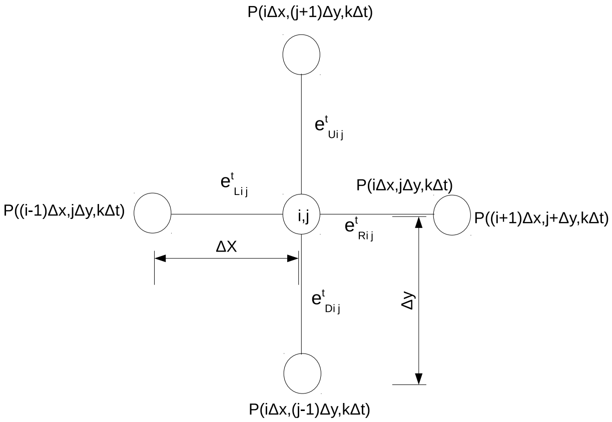

Fig. 1: Diagram1

Even more, one could extend this analisis to a n players game and use the Evolutionary Theory of Nowak. In that context, if we relate e with a particular game with payoff matrix A, and then e^{t}_{hij} is the fraction of agents in the (i,j) position that adopt strategy h at time t. The corresponding replication equation is then

$$e^{k\Delta t}_{hij}-e^{k_0\Delta t}_{hij}=e^{k_0\Delta t}_{hij}\left(f_h-\phi_h\right) \tag{9}$$

where $f_h,\ \phi_h$ are the fitness and mean fitness of strategy h.

As an example, let be the matrix A with elements $a_{hh'}$ the payoff of a Prisoner's dilemma game given in Table, then the corresponding fitness are

$$\begin{array}{lclcl} h=C\Rightarrow & f_C & = & (R)\,e^{k_0\Delta t}_{hij} & + & (S)\,e^{k_0\Delta t}_{hij} \\ h=D\Rightarrow & f_D & = & (T)\,e^{k_0\Delta t}_{hij} & + & (P)\,e^{k_0\Delta t}_{hij} \end{array}. \tag{10}$$

And the mean fitness is

$$\begin{array}{rclcl} \phi & = & \sum\limits_{\gamma} f_\gamma e^{k_0\Delta t}_{\gamma ij} e^{k_0\Delta t}_{\gamma(i+1)j} & + & \dots \\ & + & \sum\limits_{\gamma} f_\gamma e^{k_0\Delta t}_{\gamma ij} e^{k_0\Delta t}_{\gamma(i-1)j} & + & \dots \\ & + & \sum\limits_{\gamma} f_\gamma e^{k_0\Delta t}_{\gamma ij} e^{k_0\Delta t}_{\gamma i(j+1)} & + & \dots \\ & + & \sum\limits_{\gamma} f_\gamma e^{k_0\Delta t}_{\gamma ij} e^{k_0\Delta t}_{\gamma i(j-1)} & & \end{array} \tag{11}$$

Taking into account equations 10 and 11 and that $e^{k\Delta t}_{hij}-e^{k_0\Delta t}_{hij}$is the discrete form of the time derivative, the replicator equation can be rewritten [3] in matrix form as

$$\frac{dE}{dt}=\left[\Lambda\left(t\right),E\left(t\right)\right]. \tag{12}$$

Where E,Λ are two matrix with elements

$$E_{hh'}=\left(e_h e_{h'}\right)^{1/2} \tag{13}$$

and

$$\Lambda_{hh'} = \frac{1}{2}\left[\left(\sum_{k=1}^{n} a_{hk}e_k\right)(e_h e_{h'})^{1/2} - (e_h e_{h'})^{1/2}\left(\sum_{k=1}^{n} a_{hk}e_k\right)\right], \tag{14}$$

and the square brackets [] in equation 12, stands for commutation operation. It has been demonstrated that quantum game theory is a generalization of game theory an that the replicator equation 12 is equivalent to von Neumann equation

$$i\hbar\frac{d\rho}{dt} = [H, \rho] \tag{15}$$

Where ρ is the density matrix, a a self-adjoint (or Hermitian) positive-semidefinite matrix of trace one, that describes the statistical state of a quantum system. The H operator is the hamiltonian of the system. The equivalence between the replicator and von Neumann equations are given by [3]

$$E \leftrightarrow \rho,\ \Lambda \leftrightarrow -\frac{i}{\hbar}H. \tag{16}$$

Via the master equation, it can be demonstrated [4, 5, 6] that the von Neumann equation leads to a Fokker-Planck equation of the form

$$\frac{\partial e(x,y,t)}{\partial t} = -div\left[D_1(x,y,t)\, e(x,y,t)\right] + \nabla\left[D_2(x,y,t)\, e(x,y,t)\right] \tag{17}$$

where D_1 and D_2 are traditionally associated with drift and diffusion.

In this game theory context $D_1(x,y,t)$ is associated with the fitness $f(x,y,t)$ (Eq.10)and $D_2(x,y,t)$ with the mean fitness $\phi(x,y,t)$ (Eq.11).

The master equation is a first-order differential equation that describe the time evolution of the probability of the system to be in a particular set of states. Typically the master equation is given by

$$\frac{d\overrightarrow{P}}{dt} = A(t)\overrightarrow{P} \tag{18}$$

where $\overrightarrow{P}$ is a column vector of the states i , and $A(t)$ is the matrix of connections. Many physical problems in classical, quantum mechanics and other sciences, can be expressed in terms of a master equation. Examples of these are the Lindblad equation in quantum mechanics and as we mention above, the Fokker–Planck equation which describes the time evolution of a continuous probability distribution. For more hydrologically aplications of the master equation the reader may refer to [7].

We can finally enunciate the discrete spatially extended game in continuum terms. The probability of finding an agent in the position (x,y) at the time t is given by

$$\frac{\partial P(x,y,t)}{\partial t} = div\left[e(x,y,t)\nabla P\right]. \tag{19}$$

Where $e(x,y,t)$is the strategy that the player in (x,y) plays at the time t and that obeys the equation

$$\frac{\partial e(x,y,t)}{\partial t} = -div\left[D_1(x,y,t)\, e(x,y,t)\right] + \nabla^2\left[D_2(x,y,t)\, e(x,y,t)\right]. \tag{20}$$

References

[1] Perona, P. and Malik, J., 1990, Scale-space and edge detection using anisotropic diffusion, IEEE Trans. PAttern Anal. Machine Intell. Vol 12(7).

[2] Black, M. and Marimott D., 1998, Robust Anisotropic Diffusion, IEEE Trans. on image processing. Vol 7(3)

[3] Quevara, E., 2005, Quantum Replicator Dynamics, arXiv:quant-ph/0510238v7

[4] Kenkre, V. M. 2003. Memory formalism, nonlinear techniques, and kinetic equation approaches, in V. M. Kenkre and K. Lindenberg (Eds.), Modern Challenges in Statistical Mechanics, Patterns, Noise, and the interplay of Nonlinearity and Complexity, , AIP Conference Proceedings volume 658, American Institute of Physics, Melville, NY, USA.

[5] Curado, W. and Nobre, F., 2003, Derivation of nonlinear Fokker-Planck equations by means of approximations to the master equation, PHYSICAL REVIEW E 67, 021107

[6] Claussen, J., 2008, Discrete stochastic processes, replicator and Fokker-Planck equations of coevolutionary dynamics in finite and infinite populations, arXiv:0803.2443v1 [q-bio.PE]

[7] Berkowitz, B., Cortis, A., Dentz, M. and Scher, H. 2006, Modeling non-Fickian transport in geological formation as a continuous time random walk, Rev. Goephys. 44
